# Nesfatin-1_30-59_ Injected Intracerebroventricularly Increases Anxiety, Depression-Like Behavior, and Anhedonia in Normal Weight Rats

**DOI:** 10.3390/nu10121889

**Published:** 2018-12-01

**Authors:** Stephanie Gladys Kühne, Martha Anna Schalla, Tiemo Friedrich, Peter Kobelt, Miriam Goebel-Stengel, Melissa Long, Marion Rivalan, York Winter, Matthias Rose, Andreas Stengel

**Affiliations:** 1 Charité Center for Internal Medicine and Dermatology, Department for Psychosomatic Medicine, Charité-Universitätsmedizin, Corporate Member of Freie Universität Berlin, Humboldt-Universität zu Berlin and Berlin Institute of Health, 12203 Berlin, Germany; stephanie.kuehne@charite.de (S.G.K.); martha.schalla@charite.de (M.A.S.); tiemo.friedrich@charite.de (T.F.); peter.kobelt@charite.de (P.K.); miriam.stengel@helios-gesundheit.de (M.G.-S.); matthias.rose@charite.de (M.R.); 2 Department of Internal Medicine, Helios Clinic, 78628 Rottweil, Germany; 3 Department of Psychosomatic Medicine and Psychotherapy, University Hospital Tübingen, 72076 Tübingen, Germany; 4 Cognitive Neurobiology, Berlin Mouse Clinic for Neurology and Psychiatry, Humboldt University, 10117 Berlin, Germany; melissa.long@charite.de (M.L.); marion.rivalan@charite.de (M.R.); York.Winter@charite.de (Y.W.)

**Keywords:** behavior, depression, gut-brain axis, NUCB2, obesity, psychosomatic

## Abstract

Nesfatin-1 is a well-established anorexigenic peptide. Recent studies indicated an association between nesfatin-1 and anxiety/depression-like behavior. However, it is unclear whether this effect is retained in obesity. The aim was to investigate the effect of nesfatin-1_30-59_—the active core of nesfatin-1—on anxiety and depression-like behavior in normal weight (NW) and diet-induced (DIO) obese rats. Male rats were intracerebroventricularly (ICV) cannulated and received nesfatin-1_30-59_ (0.1, 0.3, or 0.9 nmol/rat) or vehicle 30 min before testing. Nesfatin-1_30-59_ at a dose of 0.3 nmol reduced sucrose consumption in the sucrose preference test in NW rats compared to vehicle (–33%, *p* < 0.05), indicating depression-like/anhedonic behavior. This dose was used for all following experiments. Nesfatin-1_30-59_ also reduced cookie intake during the novelty-induced hypophagia test (−62%, *p* < 0.05). Moreover, nesfatin-1_30-59_ reduced the number of entries into the center zone in the open field test (−45%, *p* < 0.01) and the visits of open arms in the elevated zero maze test (−39%, *p* < 0.01) in NW rats indicating anxiety. Interestingly, DIO rats showed no behavioral alterations after the injection of nesfatin-1_30-59_ (*p* > 0.05). These results indicate an implication of nesfatin-1_30-59_ in the mediation of anxiety and depression-like behavior/anhedonia under normal weight conditions, while in DIO rats, a desensitization might occur.

## 1. Introduction

Nesfatin-1 was identified in the rat hypothalamus and early on established as an anorexigenic peptide [1]. The 82-amino acid polypeptide is derived from its precursor protein nucleobindin-2 (NUCB2) by post-translational cleavage and consists of an n-terminal, middle, and C-terminal domain [2]. Expression of NUCB2/nesfatin-1 in the arcuate nucleus (Arc), the paraventricular nucleus (PVN), and the nucleus of the solitary tract (NTS) further suggested a role in the regulation of food intake [3]. Intracerebroventricular (ICV) injection of the peptide resulted in a robust reduction of dark phase food intake [4], a finding subsequently replicated for the mid fragment of nesfatin-1, nesfatin-1_30-59_ [2,5] representing the active core of nesfatin-1.

Later studies also identified the expression of NUCB2/nesfatin-1 in the periphery such as in adipose tissue [6], endocrine pancreatic beta cells [7], testis [8] and a major source in the stomach, namely in endocrine X/A-like cells of the stomach where it is co-localized with ghrelin [9]. These findings were subsequently confirmed in humans [10]. Despite the fact that this finding suggested an implication of peripheral nesfatin-1 in the regulation of food intake, the anorexigenic effect was more readily observed after central injection while peripheral application had no effect on food intake [4] or required very high doses [2], although nesfatin-1 was shown to cross the blood-brain barrier in both directions [11,12]. Later on, studies identified other effects of peripheral nesfatin-1 such as a role in glucose homeostasis [13], an anti-inflammatory action [14], and an increase of blood pressure [15] pointing towards more pleiotropic effects of the peptide, an assumption recently supported by the widespread distribution of nesfatin-1 autoradiographic signals [16], a surrogate parameter for the expression of the nesfatin-1 receptor which is still to be identified [17]. These signals were detected in the gastric mucosa, duodenum, jejunum, ileum, pancreas, adrenal gland, testis, visceral adipose tissue, heart, skeletal muscle, lung, liver and kidney as well as in the pituitary, cortex, paraventricular nucleus of the hypothalamus, area postrema, dorsal motor nucleus of the vagus nerve and cerebellum [16].

In the brain, the release of food intake-regulatory peptides is often affected by aversive situations [18], indicating an involvement of these peptides in the stress response. This was shown e.g., for bombesin [19] or ghrelin [20] or nesfatin-1, for which several links with the stress-signaling system have been observed. First, nesfatin-1’s food intake-inhibitory action is mediated by downstream corticotropin-releasing factor (CRF) receptor 2 signaling [4]. Second, ICV injection of nesfatin-1 activates CRF-positive neurons subsequently increasing circulating adrenocorticotropic hormone (ACTH) and corticosterone levels [21]. Lastly, several stressors activate brain NUCB2/nesfatin-1 signaling and also increase circulating NUCB2/nesfatin-1 levels [22,23]. These include psychological (restraint or water avoidance) [22,24], physical (abdominal surgery) [25], and immunological (injection of lipopolysaccharide) stressors [26]. 

A recent study described that circulating NUCB2/nesfatin-1 levels were significantly higher in patients with major depressive disorder compared to healthy controls [27]. Additionally, elevated plasma levels of NUCB2/nesfatin-1 have been reported in female obese inpatients with high anxiety scores resulting in a correlation between NUCB2/nesfatin-1 and self-reported anxiety [28]. However, these associations do not allow us to draw a causal conclusion. In rats, nesfatin-1 was shown to increase anxiety and fear-related behavior after an ICV injection [29], and also repeated intraperitoneal injection of nesfatin-1 increased anxiety and decreased exploratory behavior in rats [30]. However, it is unclear whether the short fragment, nesfatin-1_30-59_, also affects anxiety and depression-like behavior in rats and whether these effects are retained under conditions of obesity often leading to a reduction/loss of function of several food intake-regulatory peptides [31].

Therefore, in the present study we investigated the effects of ICV injected nesfatin-1_30-59_, proposed to be the active core of nesfatin-1, on anxiety behavior using the open field test, elevated zero maze, and light/dark box as well as on anhedonic/depression-like behavior using the sucrose preference and the novelty-induced hypophagia test in normal weight rats. To investigate whether these effects are retained under conditions of obesity, we also performed these tests in diet-induced obese (DIO) rats.

## 2. Materials and Methods 

### 2.1. Animals

For the experiments, male Sprague Dawley rats (Envigo, Germany) weighing 200–250 g were used. Rats were group housed during the acclimatization period under controlled conditions with a 12-h dark/light cycle with lights on at 6 AM, humidity of 45–65% and at a temperature of 21–23 °C with *ad libitum* access to water and standard rodent diet (D12450B, Research Diets, Inc., Jules Lane, New Brunswick, NJ, USA). Body weight and food intake were measured daily, during this time rats were handled daily to become accustomed to the investigators. Animal care and experimental procedures followed institutional ethics guidelines and were approved by the state authority for animal research (Landesamt für Gesundheit und Soziales Berlin, LaGeSo Berlin).

### 2.2. Diets 

The rats were divided into two groups: one group received a standard rodent diet (D12450B, Research Diets, Inc., Jules Lane, New Brunswick, NJ, USA, 3.9 kcal/g, 10% fat, 70% carbohydrates, 20% proteins) and the other group received a high-fat diet (D12451, Research Diets, Inc., Jules Lane, New Brunswick, NJ, USA, 4.7 kcal/g, 45% fat, 35% carbohydrates, 20% proteins). After 10 weeks rats developed DIO, a well-established animal model for obesity. Since only 50% of rats develop DIO, these rats were selected after 10 weeks as reported before [5]. The average body weight of the DIO rats at the beginning of the experiments was 426.9 ± 9.5 g, while the average body weight of the normal weight rats was 259.1 ± 3.1 g at the start of the experiments (*p* < 0.001).

### 2.3. Intracerebroventricular Cannulation

Before surgery, the rat was anesthetized with ketamine (100 mg/kg; Ketanest^TM^, Curamed, Karlsruhe, Germany) and xylazine (10 mg/kg; Rompun^TM^, 2%, Bayer, Leverkusen, Germany) as described before [32]. Then, the animal was placed in a stereotaxic apparatus and a small incision in the scalp was made to expose the bregma. After localization of the bregma, the right location to implant a guide cannula (0.8 mm posterior, 1.5 mm right lateral, and 3.5 mm ventral from bregma) was determined using the rat brain atlas [33], and a hole was drilled into the scull. The guide cannula (22-gauge, Plastics One Inc., Roanoke, VA, USA) was inserted into the right lateral ventricle and fixed on the scull with four sterile stainless-steel screws (Plastics One Inc., Roanoke, VA, USA) and dental cement (Stoelting Co., Wood Dale, IL, USA). The guide cannula was closed with a dummy cannula. To reduce postoperative pain and to avoid infection, rats received buprenorphine (0.03 mg/kg subcutaneously for three days) and enrofloxacin (2.5% ad us. vet. 0.1 ml/L in drinking water, Bayer Vital GmbH, Leverkusen, Germany).

After surgery, rats were singly housed. They had five days to recover and were handled daily with light restraint to get used to the injection procedure. The correct position of the ICV cannula was verified after the last experiment when rats were sacrificed by pentobarbital overdose. A volume of 10 µL of 0.1% toluidine blue was injected into the lateral brain ventricle as described before [32]. Correct placement of the cannula was evaluated under the microscope and indicated by spreading of the dye throughout the brain ventricular system. No rats had to be retrospectively excluded from analyses.

### 2.4. Peptide and Intracerebroventricular Injection

Rat nesfatin-1_30-59_ (Bachem AG, Weil am Rhein, Germany) was stored as a powder at –80 °C and aliquoted in sterile distilled water before the experiments. For ICV injections, nesfatin-1_30-59_ was applied at three different doses, namely 0.1, 0.3 and 0.9 nmol diluted in 5 µL sterile double distilled H_2_O to ensure the sterility of the injection. Doses were based on previous experiments investigating the effects of nesfatin-1_30-59_ on food intake [5].

On the day of the experiment, lightly hand restrained rats were slowly (over 15 s followed by 60 s time to drain from the ventricle) injected ICV with vehicle (5 µL sterile H_2_O) or nesfatin-1_30-59_ (0.1, 0.3 or 0.9 nmol in 5 µL sterile H_2_O) using a 28-gauge cannula (Plastics One Inc., Roanoke, VA, USA) 1 mm longer than the guide cannula and connected to a 25-µL Hamilton syringe by a PE-50 catheter (Intramedic Polyethylene Tubing, Clay Adams, Parsippany, NJ, USA). Since the dose of 0.3 nmol/rat exerted the most pronounced effect in the sucrose preference test, this dose was used for all subsequent experiments.

### 2.5. Experimental Design and Procedures

To assess anxiety, exploratory behavior, depression-like behavior, and anhedonia, several well-established tests were performed in normal weight as well as DIO rats. Animals were ICV injected with vehicle or nesfatin-1_30-59_ at the beginning of the dark phase as the main effect of nesfatin-1 on food intake was observed during this photoperiod [4], and were tested 30 min later. All animals performed at least two tests and rats had at least three days to recover in between the tests. To reduce the number of animals, an in-between test crossover design was used. All experiments were conducted at the beginning of the dark phase.

#### 2.5.1. Sucrose Preference Test

The sucrose preference test examines anhedonic behavior, a component of depression-like behavior [34] and was performed as follows: Three days before the experiment animals received a 1% sucrose solution for 48 h additionally to their regular drinking water. At 24 h before the experiment, the sucrose solution was removed. On the day of the experiment, normal weight rats were ICV injected with nesfatin-1_30-59_ (0.1, 0.3 or 0.9 nmol/rat) or vehicle and 30 min later rats had access to the 1% sucrose solution in addition to regular water and solid diet. Water and sucrose intakes were monitored by an automated food intake monitoring system (BioDAQ, Research Diets Inc., Jules Lane, New Brunswick, NJ, USA) as established before [35].

Briefly, food and water were placed on microbalances that weigh the food and water hoppers every second (± 0.01 g) and detect “not eating/drinking” as weight stable and “eating/drinking” as weight unstable. Feeding/drinking bouts (changes in stable weight before and after a bout) are recorded with a start time, duration and amount consumed. Bouts are separated by an inter-bout interval (IBI). Meals consist of one or more bouts separated by an inter-meal interval (IMI) defined as 15 min with a minimum meal size of 0.1 g as in our previous study [36]. Rats were habituated to the system for five days before the test started.

Water and sucrose intakes were monitored in an automated fashion for 1 h and the “sucrose to total fluid intake” ratio determined. A decreased sucrose intake is a surrogate of anhedonic behavior [37]. 

#### 2.5.2. Novelty-Induced Hypophagia

The novelty-induced hypophagia test assesses anxious and depression-like behavior [38]. We used an adapted form as described before [29]. During a training period, rats received a palatable snack (HoneyMaid^TM^ Graham Cracker Crumbs, Nabisco, East Hanover, NJ, USA) and water *ad libitum* at the beginning of the dark phase for 30 min for five consecutive days. Food intake was assessed in an automated fashion (BioDAQ, Research Diets Inc., Jules Lane, New Brunswick, NJ, USA) as described above to assess a stable baseline food intake.

On the day of the experiment, rats were ICV injected with nesfatin-1_30-59_ (0.3 nmol/rat) or vehicle, placed back in their home cages and, 30 min later, moved in a novel cage without bedding or enrichment inducing novelty stress. Here, animals had access to the familiar palatable snack and water *ad libitum* for 30 min and food intake microstructure was assessed for another 30 min.

#### 2.5.3. Open Field Test

The open field test exposes the rat to a new and unfamiliar environment [39] and assesses explorative behavior as a surrogate for anxiety. The test was performed in a 50 × 50 cm white polyvinylchloride box with a black floor. This box can be divided into a center zone and an outer zone (Figure S1). A camera records duration and entries into the center zone as well as the locomotion of the animal including the total distance crossed. A long total distance and high locomotion along with a long duration in center zone and multiple entries into the center zone are interpreted as explorative behavior, whereas a reduction of these parameters reflects anxious behavior.

On the day of the experiment, rats were ICV injected with nesfatin-1_30-59_ (0.3 nmol/rat) or vehicle and 30 min later placed in the center of the open field box. Behavior was recorded for 5 min and analyzed using a connected software (Biobserve GmbH, Bonn, Germany). After every test, the apparatus was cleaned with 5% ethanol.

#### 2.5.4. Elevated Zero Maze

The elevated zero maze is a well-established tool to assess explorative behavior and anxiety [40] and is a further development of the elevated plus maze with the advantage of a higher sensitivity [41]. It consists of a zero-shaped elevated platform with the North and South wings being open arms, while the East and West wings are closed (Figure S1). A video camera records the total track length, the time and number of entries into open and closed arms and the average velocity. Animals are considered as being in the open/closed area when all four paws are in the respective arm. A high average track length and velocity represent high locomotor activity indicative of explorative behavior. Likewise, the time in open arms indicates explorative behavior, whereas the time in closed arms reflects anxious behavior. Besides track length, direct locomotor activity was not measured outside of this test, since previous studies indicated that nesfatin-1 ICV has no effect on locomotion [4,42].

On the day of the experiment, rats were ICV injected with nesfatin-1_30-59_ (0.3 nmol/rat) or vehicle, 30 min later placed in the open arm of the elevated zero maze and their behavior was recorded for 5 min and analyzed using a connected software (Biobserve GmbH, Bonn, Germany). After every test, the apparatus was cleaned with 5% ethanol.

#### 2.5.5. Light/Dark Box

The light/dark box is another test to assess the rats’ explorative behavior and anxiety [43]. The apparatus consists of two compartments, one dark and one light, connected by a small hole (Figure S1). Anxious animals tend to spend more time in the dark compartment [44]. As in the experiments described above, a camera records the movements of the animal.

On the day of the experiment, rats were ICV injected with nesfatin-1_30-59_ (0.3 nmol/rat) or vehicle, placed in the bright compartment 30 min later and their behavior, including latency to cross to the dark compartment, entries into, and duration in both compartments was recorded for 10 min and analyzed using a connected software (Biobserve GmbH, Bonn, Germany). After every test, the apparatus was cleaned with 5% ethanol.

### 2.6. Statistical Analysis

The Kolmogorov-Smirnov test was used to test the distribution of the data. Normally distributed data were assessed using the *t*-test, whereas for non-normally distributed data the Mann–Whitney-U test was applied. For the comparison of multiple groups, a one-way ANOVA followed by Tukey *post hoc* test was applied. For better comparability, all data were displayed as mean ± sem. Differences between groups were considered significant when *p* < 0.05 (SigmaStat 3.1., Systat Software, San Jose, CA, USA).

## 3. Results

### 3.1. Nefatin-1_30-59_ Injected Intracerebroventricularly Induced Anhedonic and Depression-Like Behavior in Normal Weight but Not DIO Rats

**Figure 1:**
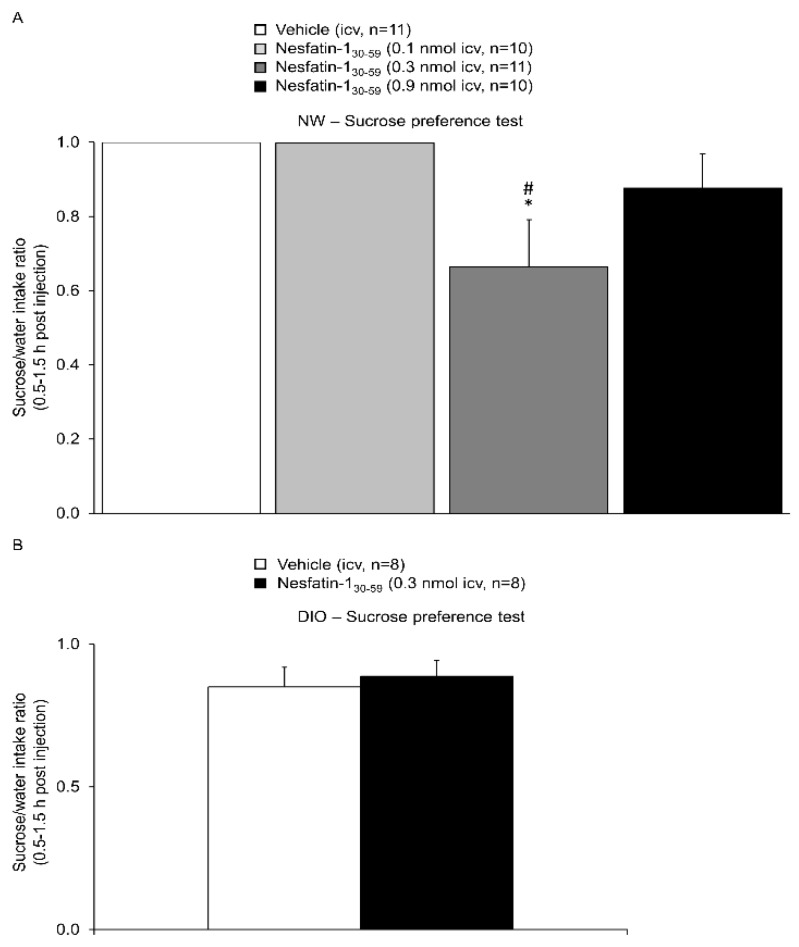
**Nesfatin-1_30-59_ decreased sucrose preference in normal weight but not in diet-induced obese rats.** (**A**) Intracerebroventricularly cannulated normal weight rats were injected with vehicle (5 µL H_2_O, *n* = 11) or nesfatin-1_30-59_ (0.1 nmol/rat, *n* = 10, 0.3 nmol/rat, *n* = 11, or 0.9 nmol/rat, *n* = 10, in 5 µL H_2_O) at the beginning of the dark phase and water as well as sucrose intake assessed between 0.5 and 1.5 h post injection. Nesfatin-1_30-59_ reduced the sucrose/water intake ratio at a dose of 0.3 nmol/rat; this dose was used for all further analyses. (**B**) Diet-induced obese rats injected with nesfatin-1_30-59_ (0.3 nmol/rat in 5 µL H_2_O, *n* = 8) did not show significant differences in sucrose preference compared to diet-induced obese rats injected with vehicle (5 µL H_2_O, *n* = 8). Data were not normally distributed, for better comparability all data are expressed as mean ± SEM. Data were analyzed using one way ANOVA (**A**) or Mann–Whitney-U test (**B**). Abbreviations: DIO, diet-induced obesity; NW, normal weight. * *p* < 0.05 vs. vehicle; # *p* < 0.05 vs. Nesfatin-1_30-59_ 0.1 nmol.

Nesfatin-1_30-59_ injected in normal weight rats ICV at a dose of 0.3 nmol significantly reduced the sucrose/water intake ratio to 0.66 ± 0.13 (*n* = 11) compared to vehicle (1.00 ± 0.00, *n* = 11) and nesfatin-1_30-59_ at a dose of 0.1 nmol (1.00 ± 0.00, *p* = 0.03; *n* = 10), while compared to nesfatin-1_30-59_ at a dose of 0.9 nmol (0.88 ± 0.09, *n* = 10) did not reach significance (*p* = 0.30; Figure 1) reflective of an anhedonic effect. Based on this experiment the dose of 0.3 nmol nesfatin-1_30-59_ was used for all subsequent experiments. In contrast, when nesfatin-1_30-59_ was injected at 0.3 nmol in DIO rats, no difference in sucrose intake was observed as reflected by a similar sucrose/water intake ratio in nesfatin-1_30-59_ and vehicle treated rats (*p* = 0.69; *n* = 8/group, Figure 1).

**Figure 2:**
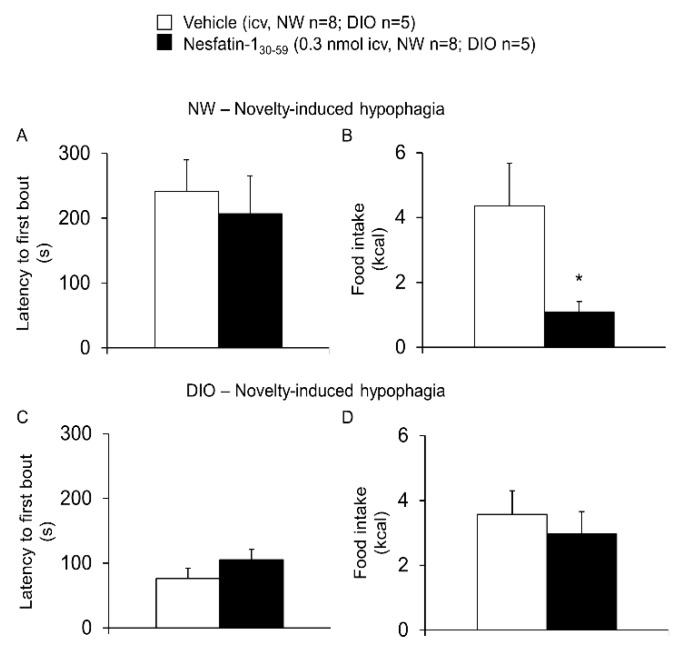
**Nesfatin-1_30-59_ increased novelty-induced hypophagia in normal weight but not in diet-induced obese rats.** Intracerebroventricularly cannulated normal weight or diet-induced obese rats were injected with vehicle (5 µL H_2_O, *n* = 8 and *n* = 5, respectively) or nesfatin-1_30-59_ (0.3 nmol/rat in 5 µL H_2_O, *n* = 8 and *n* = 5, respectively) at the beginning of the dark phase and 30 min later placed into a novel cage without bedding or enrichment. The latency to approach the food and the intake of a palatable snack was assessed for another 30 min. In normal weight rats, the latency to approach the food did not differ between the nesfatin-1_30-59_ and vehicle animals (**A**), whereas the total food intake (g) was significantly decreased in nesfatin-1_30-59_ injected rats (**B**). In diet-induced obese rats, neither the latency to approach the food (**C**) nor the food intake (**D**) was significantly affected by the nesfatin-1_30-59_ injection (0.3 nmol/rat in 5 µL H_2_O) compared to vehicle (5 µL H_2_O). Data were normally distributed and are expressed as mean ± sem. Data have been analyzed by *t*-test. Abbreviations: DIO, diet-induced obesity; NW, normal weight. * *p* < 0.05 vs. vehicle.

In the novelty-induced hypophagia test normal weight rats treated with nesfatin-1_30-59_ (0.3 nmol/rat) showed a significantly reduced intake of the palatable food compared to vehicle (1.09 ± 0.32 vs. 4.36 ± 1.31 kcal, *p* = 0.04), while the latency to approach the food was not significantly altered (207.0 ± 58.4 vs. 241.3 ± 48.8 s, *p* = 0.68; *n* = 8/group, Figure 2). Overall food intake (24-h) of the standard rodent diet was not significantly different between the nesfatin-1_30-59_ and vehicle group before (58.3 ± 5.7 vs. 65.6 ± 3.7 kcal, *p* = 0.73) and after (60.5 ± 1.8 vs. 55.5 ± 3.1 kcal, *p* = 0.07) the novelty-induced hypophagia test. In DIO rats, none of these parameters was significantly altered during the novelty-induced hypophagia test by 0.3 nmol nesfatin-1_30-59_ compared to the vehicle (*p* = 0.61 and *p* = 0.64; *n* = 5/group, Figure 2). Similarly, the 24-h food intake of the high-fat diet was not different between the nesfatin-1_30-59_ and vehicle group before (50.8 ± 2.3 vs. 59.6 ± 4.0 kcal, *p* = 0.13) and after (67.7 ± 8.5 vs. 45.8 ± 9.5 kcal, *p* = 0.29) the novelty-induced hypophagia test. 

### 3.2. Nesfatin-1_30-59_ Injected Intracerebroventricularly Induced Anxious Behavior in Normal Weight but Not DIO Rats

**Figure 3:**
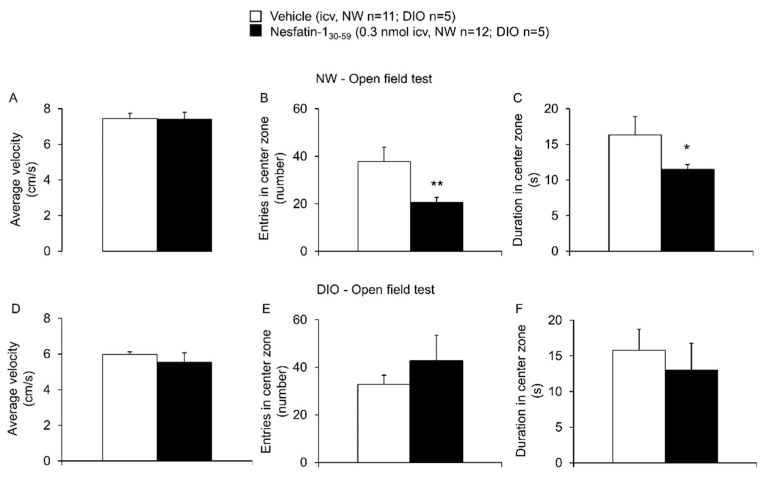
**Nesfatin-1_30-59_ induced anxiety in the open field test in normal weight but not in diet-induced obese rats.** Intracerebroventricularly cannulated normal weight or diet-induced obese rats were injected with vehicle (5 µL H_2_O, *n* = 11 and *n* = 5, respectively) or nesfatin-1_30-59_ (0.3 nmol/rat in 5 µL H_2_O, *n* = 12 and *n* = 5, respectively) at the beginning of the dark phase and 30 min later placed in a box with a center and an outer zone. Behavior including velocity ((**A**): normal weight; (**D**): diet-induced obesity), entries in the center zone ((**B**): normal weight; (**E**): diet-induced obesity) and duration in the center zone ((**C**): normal weight; (**F**): diet-induced obesity) was assessed for 5 min using a computer-supported technique. While the number of entries in center zone and the duration in the center zone were significantly decreased in normal weight nesfatin-1_30-59_ injected rats compared to vehicle, no significant changes were observed in diet-induced obese rats. Data were distributed normally except for the parameter entries in center zone in the normal weight group treated with nesfatin-1_30-59_. For better comparability, all data are expressed as mean ± SEM. Data were analyzed using the Mann–Whitney-U test (entries in center zone in the normal weight group) or *t*-test (all other data). Abbreviations: DIO, diet-induced obesity; NW, normal weight. * *p* < 0.05 and ** *p* < 0.01 vs. vehicle.

Nesfatin-1_30-59_ injected in normal weight rats ICV at a dose of 0.3 nmol (*n* = 12) significantly reduced the entries into the center zone (20.6 ± 2.0 vs. 37.8 ± 6.1, *p* = 0.008) and reduced the duration spent in this zone (11.5 ± 0.7 vs. 16.3 ± 2.6 s, *p* = 0.04), whereas the average velocity in the open field test was not altered compared to vehicle (7.4 ± 0.4 vs. 7.4 ± 0.5 cm/s, *p* = 0.47; *n* = 11, Figure 3). The overall distance crossed was not different between the nesfatin-1_30-59_ and vehicle groups (22.3 ± 1.2 vs. 22.6 ± 0.9 m, *p* = 0.82). None of these parameters was significantly altered by 0.3 nmol nesfatin-1_30-59_ in DIO rats when compared to vehicle (*p* = 0.47, *p* = 0.63 and *p* = 0.50; *n* = 5/group, Figure 3, overall distance crossed: 16.9 ± 1.6 vs. 18.5 ± 0.7 m, *p* = 0.41). 

**Figure 4:**
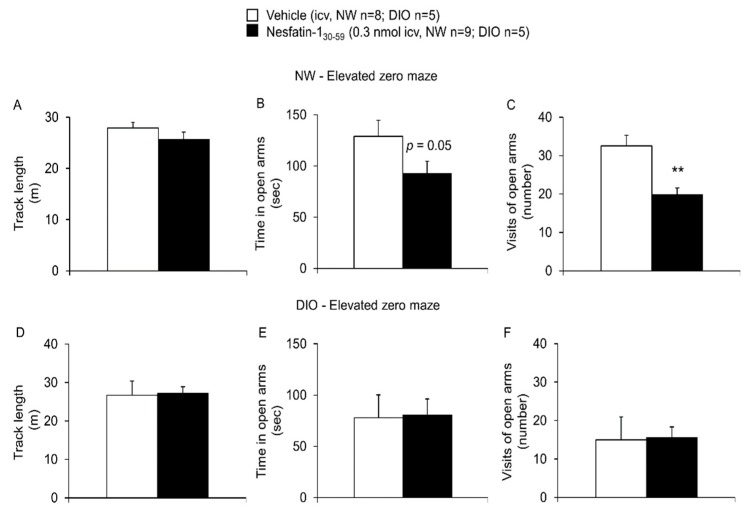
**Nesfatin-1_30-59_ induced anxiety in the elevated zero maze in normal weight but not in diet-induced obese rats.** Intracerebroventricularly cannulated normal weight or diet-induced obese rats were injected with vehicle (5 µL H_2_O, *n* = 8 and *n* = 5, respectively) or nesfatin-1_30-59_ (0.3 nmol/rat in 5 µL H_2_O, *n* = 9 and *n* = 5, respectively) at the beginning of the dark phase and 30 min later placed on a zero-shaped, elevated platform with two closed and two open zones. Behavior including visits of open arms ((**A**): normal weight; (**D**): diet-induced obesity), time in open arms ((**B**): normal weight; (**E**): diet-induced obesity) and track length ((**C**): normal weight; (**F**): diet-induced obesity) were assessed for 5 min using a computer-supported technique. While a tendency in the time in open arms (*p* = 0.05) and a significant reduction of the number of visits in open arms was observed in nesfatin-1_30-59_ injected rats compared to vehicle, none of the parameters were affected in diet-induced obese rats. All data were distributed normally except for the time in open arms and visits of open arms in the DIO/vehicle group. For better comparability, all data are expressed as mean ± SEM. Data have been analyzed using the Mann–Whitney-U test (time in open arms and visits of open arms in DIO) or *t*-test (all other data). Abbreviations: DIO, diet-induced obesity; NW, normal weight. ** *p* < 0.01 vs. vehicle.

In the elevated zero maze, nesfatin-1_30-59_ injected ICV at 0.3 nmol/rat in normal weight rats (*n* = 9) reduced the visits of the open arms (19.8 ± 1.8 vs. 32.5 ± 2.8, *p* = 0.002) and tended to decrease the time in open arms (92.7 ± 11.9 vs. 128.9 ± 15.6 s, *p* = 0.05), whereas the overall track length crossed was not different compared to vehicle (25.6± 1.4 vs. 27.9 ± 1.1 m, *p* = 0.14; *n* = 8, Figure 4). Again, none of these parameters was altered by 0.3 nmol nesfatin-1_30-59_ in DIO rats when compared to vehicle (*p* = 0.94, *p* = 0.93 and *p* = 0.93; *n* = 5/group, Figure 4).

**Figure 5:**
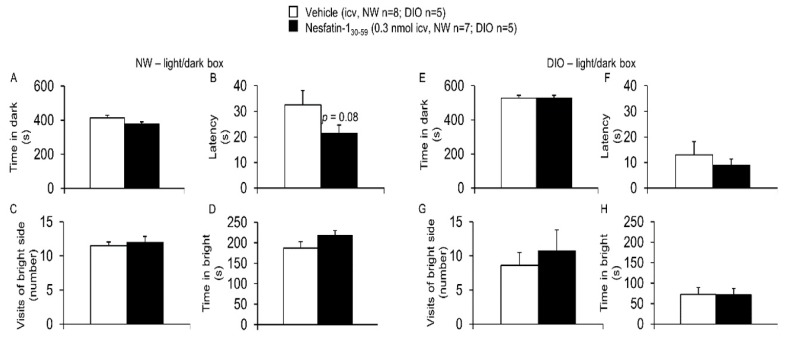
**Nesfatin-1_30-59_ neither affected the explorative behavior in the light/dark box in normal weight nor in diet-induced obese rats.** Intracerebroventricularly cannulated normal weight or diet-induced obese rats were injected with vehicle (5 µL H_2_O, *n* = 8 and *n* = 5, respectively) or nesfatin-1_30-59_ (0.3 nmol/rat in 5 µL H_2_O, *n* = 7 and *n* = 5, respectively) at the beginning of the dark phase and 30 min later placed in the bright compartment of the light/dark box. Behavior including time in dark ((**A**): normal weight; (**E**): diet-induced obesity), latency to the first entry into the black compartment ((**B**): normal weight; (**F**): diet-induced obesity), the number of visits of the bright side ((**C**): normal weight; (**G**): diet-induced obesity) and the time in bright side ((**D**): normal weight; (**H**): diet-induced obesity) was assessed for 10 min using a computer-supported technique. None of the parameters were affected by intracerebroventricularly injected nesfatin-1_30-59_ in any group. All data in the normal weight group were distributed normally. In the DIO group, the parameter latency was not distributed normally in the vehicle group. For better comparability, all data are expressed as mean ± SEM. Data have been analyzed using the Mann–Whitney-U test (latency in DIO) or *t*-test (all other data). Abbreviations: DIO, diet-induced obesity; NW, normal weight. *p* > 0.05.

Lastly, nesfatin-1_30-59_ injected in normal weight rats ICV at a dose of 0.3 nmol (*n* = 7) did not significantly alter the time in dark (381.9 ± 12.2 vs. 413.0 ± 15.9 s, *p* = 0.10), the latency to cross to the dark compartment (21.5 ± 3.2 vs. 32.6 ± 5.6 s, *p* = 0.08), the number of visits of the bright side (12.0 ± 0.9 vs. 11.5 ± 0.6, *p* = 0.33), and the time in the bright side (218.1 ± 12.2 vs. 187.0 ± 15.9 s, *p* = 0.10) during the light/dark box compared to vehicle treated rats (*n* = 8, Figure 5). Similarly, none of these parameters was significantly altered in DIO rats by nesfatin-1_30-59_ (0.3 nmol/rat, ICV) compared to vehicle (*p* = 0.97, *p* = 0.57, *p* = 0.63 and *p* = 0.97; *n* = 5/group, Figure 5). 

## 4. Discussion

The current findings indicate that nesfatin-1_30-59_ increases anxiety, depression-like behavior, and anhedonia in normal weight rats. However, these anxiogenic/anhedonic effects could not be observed in DIO animals.

The present study further corroborates the assumption of nesfatin-1_30-59_ being the active core of full-length nesfatin-1 as suggested before for the food intake-regulatory effect [2]. However, it has to be noted that higher doses are necessary for nesfatin-1_30-59_ (1.1 µg) compared to full-length nesfatin-1 (0.24 µg) to exert an anxiogenic action as observed before for the anorexigenic effect [5]. This might be due to differential receptor binding and/or activation, a hypothesis to be further investigated after the identification of the receptor. Moreover, the dose-dependent effect of nesfatin-1_30-59_ on sucrose preference displayed a U-shaped relation with 0.3 nmol/rat being the most effective dose. Whether antagonistic effects or supraphysiological stimulation of the receptor contributes to this U-shaped curve will have to be further investigated.

In the elevated zero maze, in normal weight rats injected with nesfatin-1_30-59_, the number of entries into the open arms was significantly reduced compared to controls, an observation giving rise to anxious behavior. This finding is consistent with a previous study showing that full-length nesfatin-1 reduces the number of entries into the open arms and the percent time spent in open arms of an elevated plus maze [29], thereby extending the finding to the elevated zero maze which has been suggested to be beneficial to assess anxiety as rats [40]. The anxiogenic effect of the peptide was further supported by the behavioral patterns observed in the open field test with lesser entries into the center zone and less time spent in this zone. These observations extend the findings of a previous study where high doses of nesfatin-1 (2, 4, and 8 µg/day) injected daily intraperitoneally over a period of three weeks led to a decreased moving distance, duration in center zone and frequency in rearing and grooming in the open field test [30]. Interestingly, nesfatin-1_30-59_, injected at the beginning of the dark phase, did not significantly alter behavior in the light/dark box, another well-established test to assess anxious behavior in rats [43]. This discrepancy might be associated with the notion that nesfatin-1 acts in a photosensitive manner with a robust action in the dark photoperiod, whereas no effect was observed during the light phase [4,42]. Moreover, also the initial study on nesfatin-1, although the experiments were conducted between 9 A.M. and 12 P.M., tested the food intake-modulating effects of the peptide under low illumination of 30–40 lx [1]. Lastly, since rodents as nocturnal animals are more active during the dark [45,46], behavioral tests should be performed during this photoperiod under conditions of low illumination. While the light/dark box intrinsically has a brightly lit zone, the other two tests (elevated zero maze and open field test) were performed under dimmed light conditions.

Moreover, nesfatin-1_30-59_ also induced anhedonic/depression-like behavior as indicated by reduced food intake of a palatable snack under novelty conditions, a.k.a. hyponeophagia, a finding in line with previous data on full-length nesfatin-1 [29]. The novelty-induced hypophagia test is a sensitive tool to evaluate anxiety and depression-like behavior that has been used to describe the anxiogenic or anxiolytic effects of drugs, such as antidepressants [47]. Likewise, nesfatin-1_30-59_ reduced the amount of sucrose consumed as reflected in a decreased sucrose/water intake ratio, a finding indicative of increased anhedonia characteristics for depressive disorder [48]. Since circulating NUCB2/nesfatin-1 levels have been reported to be elevated in patients with depression [49] or to be correlated with reported levels of depressive behavior [50] along with the finding that also high doses of nesfatin-1 injected intraperitoneally induce anhedonia in rats [51], peripheral nesfatin-1 might well be involved in the development and/or maintenance of depressive symptoms such as reduced appetite [27].

It is unlikely that the effects observed in these tests occur subsequently to the anorexigenic effect of nesfatin-1_30-59_. First, the number of snacks consumed in the home cage—unlike in the novel cage—was reported before to be not decreased after the injection of full-length nesfatin-1 [29]. Second, the anorexigenic effect was shown to have a delayed onset at 2 or 4 h after ICV injection of full length or mid fragment nesfatin-1, respectively [4,5], while the anhedonic effect was observed within the first-hour post injection. Lastly, the lack of difference in latency to reach for the familiar palatable food observed in the present study may indicate a similar motivation to approach a familiar object although placed in a new environment. Therefore, this effect is considered a specific anhedonic action of the peptide as a part of depression-like behavior, while an anxiogenic component might also contribute to the effect.

Interestingly, in contrast to the observed effects in normal weight rats, nesfatin-1_30-59_ injected ICV did not induce behavioral alterations in DIO rats in neither of the anxiety—(elevated zero maze, open field, light/dark box) or anhedonia/depression-like—(novelty-induced hypophagia, sucrose preference test) assessing tools. Nesfatin-1 is produced not only centrally but also in peripheral tissues such as adipose tissue [6] with an upregulation under conditions of obesity [6,52]. In DIO rats, the amount of adipose tissue is greatly increased, likely leading to elevated levels of NUCB2/nesfatin-1, a hypothesis corroborated by the correlation of NUCB2/nesfatin-1 with the body mass index in humans [6,53]. Since the gastric expression of NUCB2/nesfatin-1 is elevated also with increasing body mass index, this source is also likely to contribute to the high circulating levels of the peptide. Since nesfatin-1 can cross the blood-brain-barrier [11,12], it can be hypothesized that these higher NUCB2/nesfatin-1 levels can also exert central anxiogenic effects. Noteworthy chronic peripheral injections of nesfatin-1 were also shown to modulate anxiety in rats. However, the hypothesis of central effects of peripherally secreted nesfatin-1 as well as a central desensitization of nesfatin-1 signaling under conditions of obesity should be further investigated.

Centrally, the higher nesfatin-1 levels could stimulate the PVN known to secrete CRF since nesfatin-1 was shown to activate CRF positive neurons in the PVN as assessed using phospho (p)-ERK1/2 [54] likely leading to an upregulated expression of CRF mRNA [55] and CRF protein [56] which subsequently results in elevated circulating levels of adrenocorticotropic hormone (ACTH) and corticosterone [21] and may result in an increased stress response. Whether DIO rats display chronically elevated NUCB2/nesfatin-1 levels associated with increased corticosterone concentrations warrants further investigation. This chronic stimulation might lead to desensitization towards stressful and anxious situations in line with a study reporting that chronically increased glucocorticoid signaling in the hypothalamus does not induce a hyperactivity of the hypothalamus-pituitary-adrenal axis [57]. Whether a nesfatin-1 desensitization plays a role in the lack of effect of nesfatin-1_30-59_ on anxiety and anhedonia under conditions of DIO will have to be further investigated. Interestingly, the anorexigenic effect of nesfatin-1 [1] and nesfatin-1_30-59_ [2] is exerted in a leptin-independent manner possibly giving rise to different routes of downstream signaling mediating the anorexigenic or anxiogenic effects. Whether different brain areas are recruited by nesfatin-1_30-59_ under conditions of DIO could be investigated by Fos immunohistochemistry in future studies.

In summary, nesfatin-1_30-59_ injected ICV exerts an anxiogenic and anhedonic/depression-like effect under normal weight but not DIO conditions. Further investigations are needed to explore whether a desensitization of the receptor (for the dose tested in normal weight animals) in DIO rats contributes to the missing response or whether a complete resistance exists which should be tested using higher doses. Lastly, an alteration of downstream mediators might play a role in this lack of effect in obese rats which will have to be further investigated.

## Supplementary Materials

The following are available online at http://www.mdpi.com/2072-6643/10/12/1889/s1, Figure S1: Open field test, elevated zero maze and light/dark box.
