# Supplementary material for: Nesfatin-130-59 Injected Intracerebroventricularly Increases Anxiety, Depression-Like Behavior, and Anhedonia in Normal Weight Rats
Source: Nutrients. 2018 Dec 1;10(12):1889. doi: 10.3390/nu10121889 (PMC6315806; doi:10.3390/nu10121889)
Supplement: Supplementary file 1 [file nutrients-10-01889-s001.pdf]

A B C

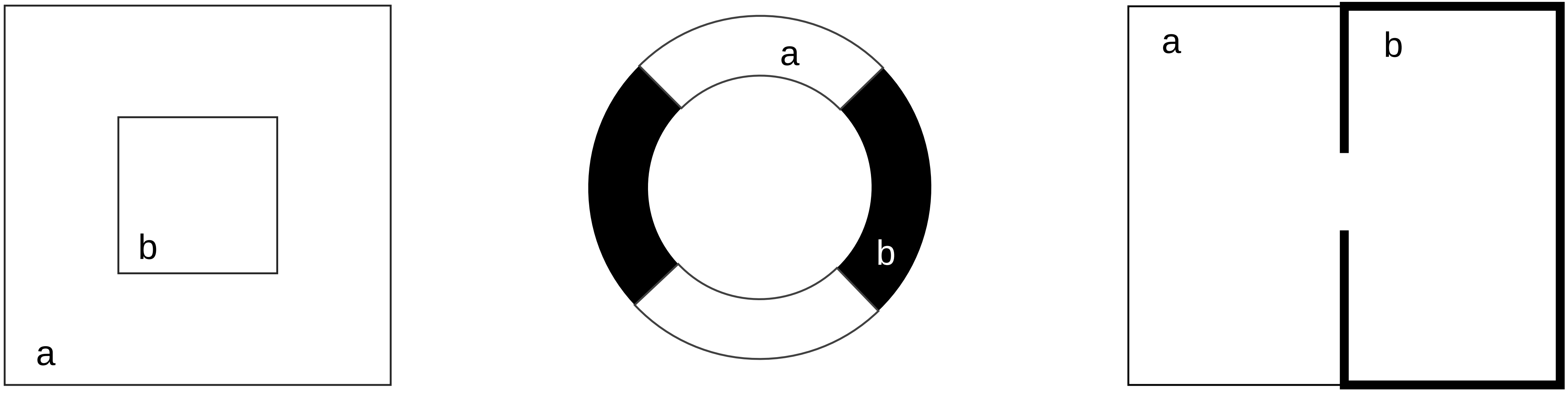

a = outer zone
b = inner zone

open field test

a = open arm
b = closed arm

elevated zero maze

a = light box
b = dark box

light/dark box
